# Supplementary material for: Digital Gene Expression Analysis of Epithelioid and Sarcomatoid Mesothelioma Reveals Differences in Immunogenicity
Source: Cancers (Basel). 2021 Apr 7;13(8):1761. doi: 10.3390/cancers13081761 (PMC8067687; doi:10.3390/cancers13081761)
Supplement: Supplementary file 1 [file cancers-13-01761-s001.zip › cancers-1141516 Supplementary figure.docx]

Supplementary Material: Digital Gene Expression Analysis of Epithelioid and Sarcomatoid Mesothelioma Reveals Differences in Immunogenicity

Luka Brcic, Alexander Mathilakathu, Robert F. H. Walter, Michael Wessolly, Elena Mairinger, Hendrik Beckert, Daniel Kreidt, Julia Steinborn, Thomas Hager, Daniel C. Christoph, Jens Kollmeier, Thomas Mairinger, Jeremias Wohlschlaeger, Kurt Werner Schmid, Sabrina Borchert and Fabian D. Mairinger


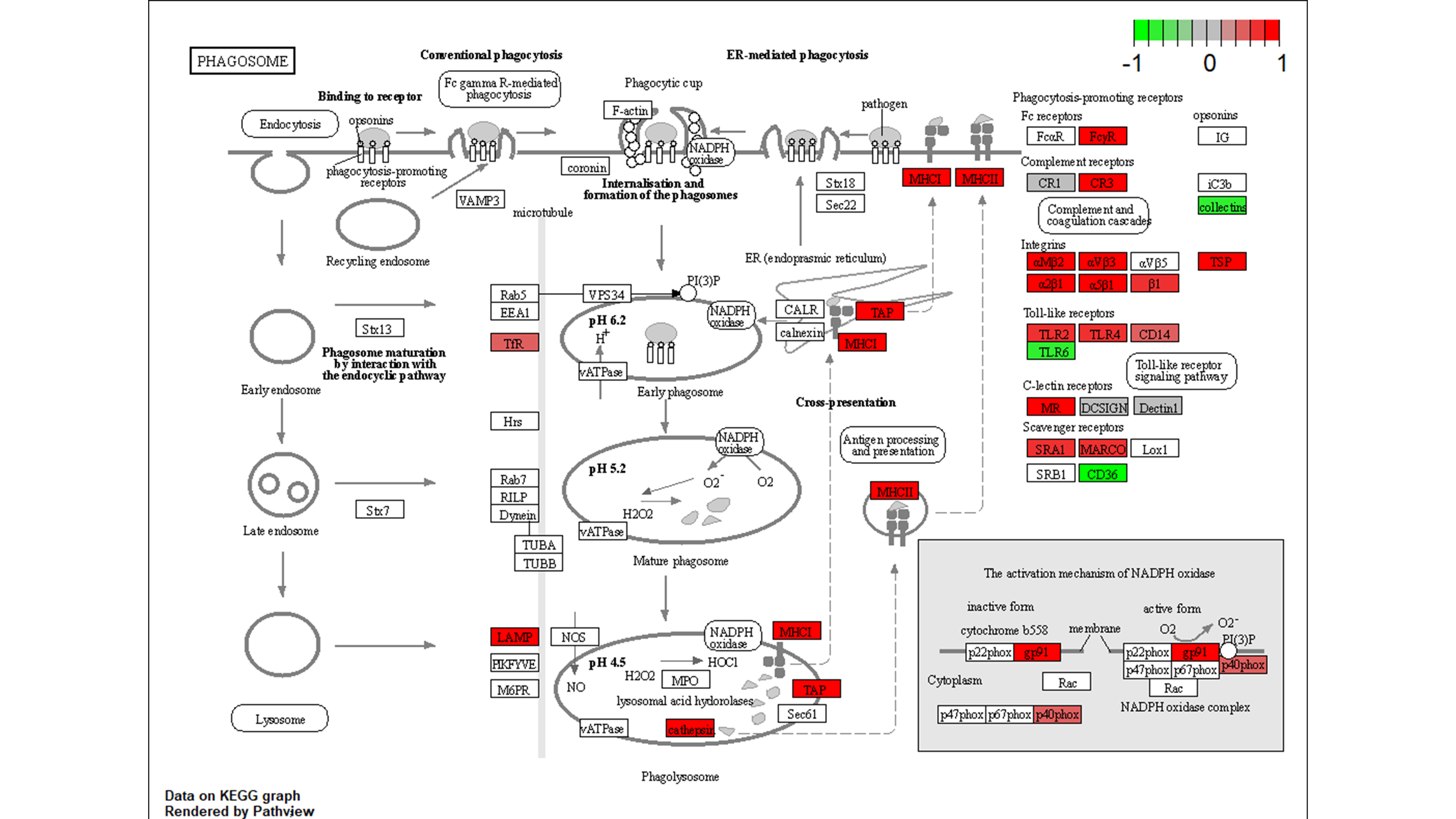


**Figure S1:** Gene set enrichment analysis of differential expressed genes between EMM and SMM involved in phagosome pathway.


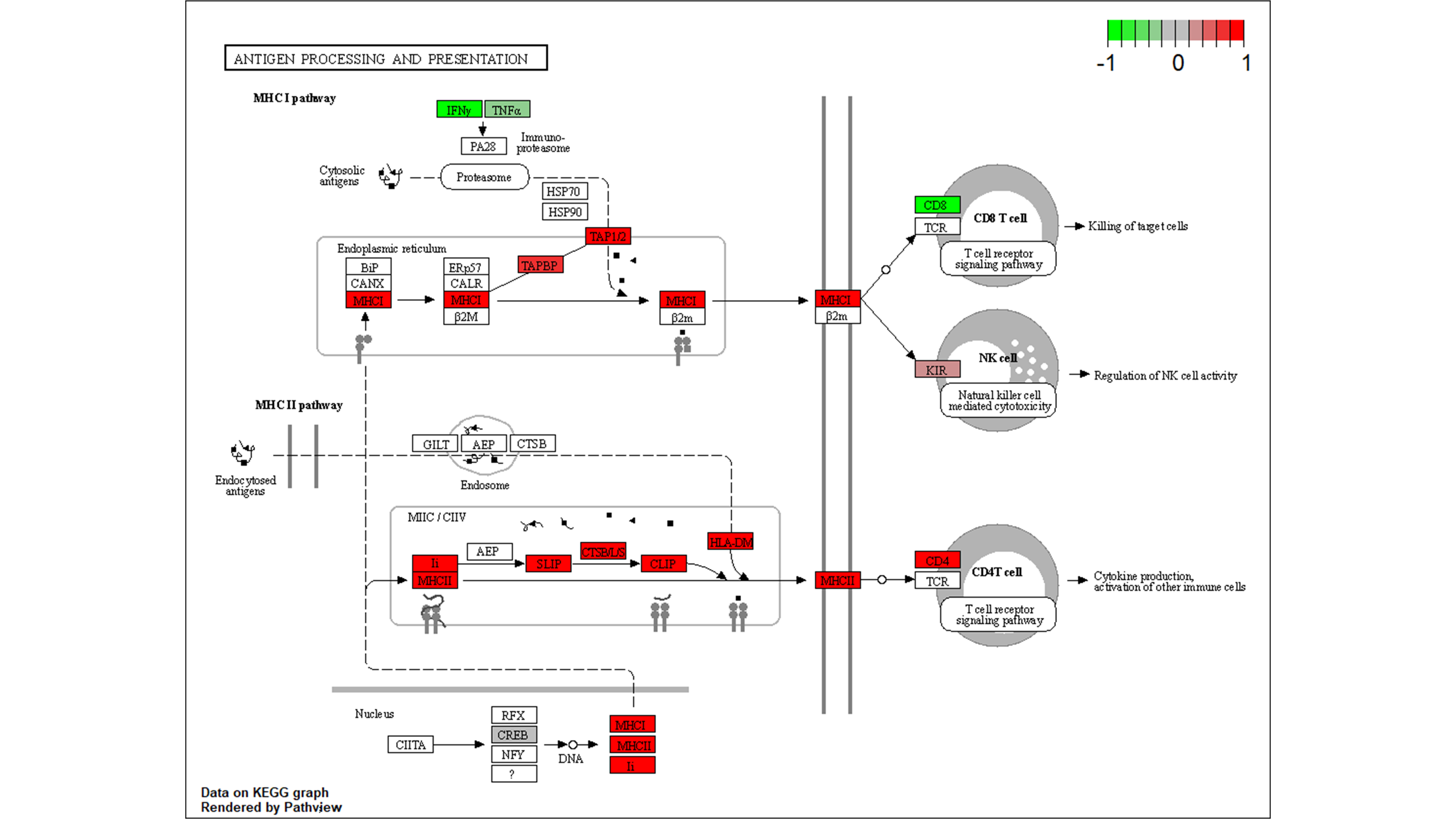


**Figure S2:** Gene set enrichment analysis of differential expressed genes between EMM and SMM involved in antigen processing and presentation pathway**.**


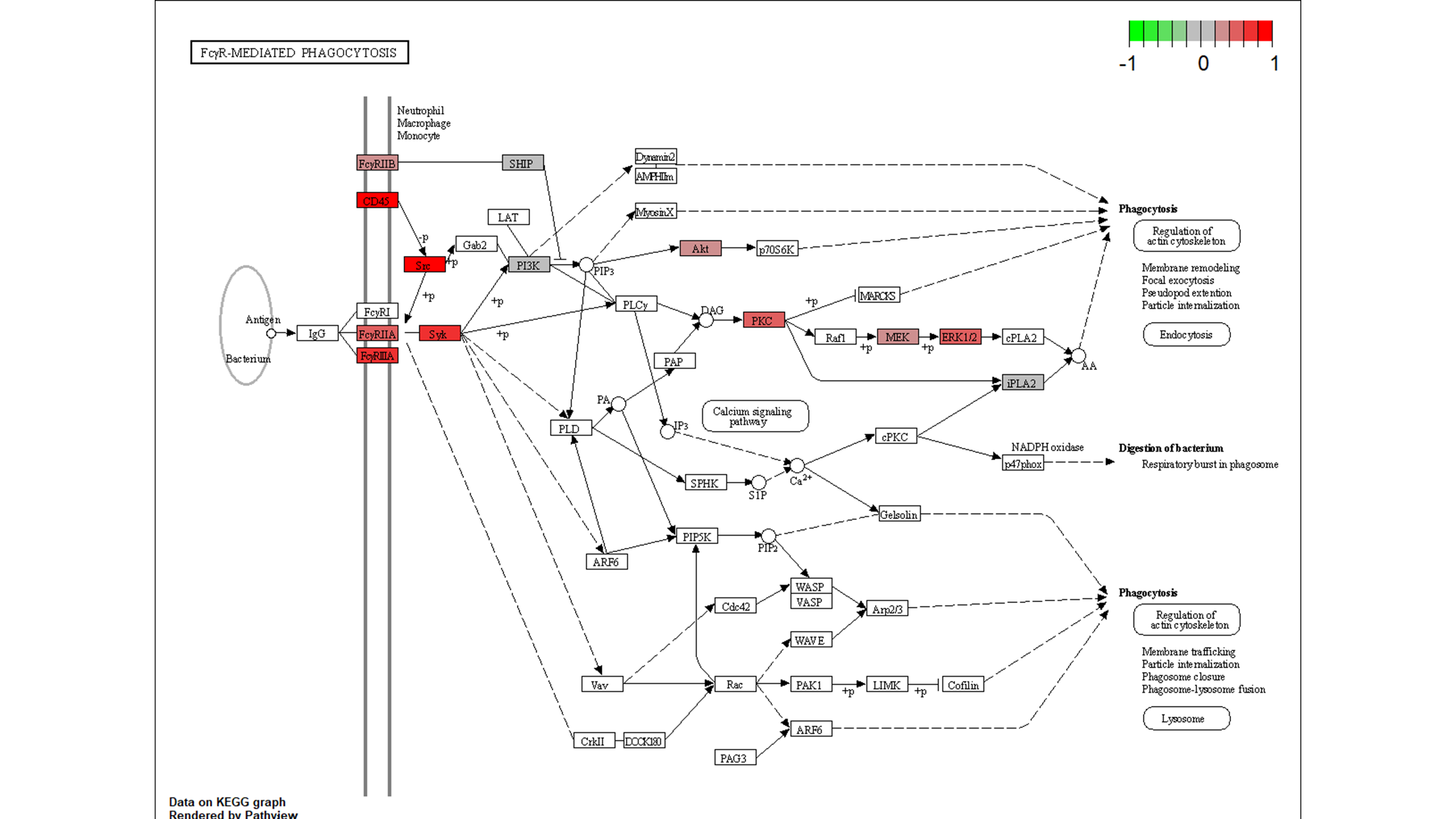


**Figure S3:** Gene set enrichment analysis of differential expressed genes between EMM and SMM involved in Fc gamma R-mediated phagocytosis.


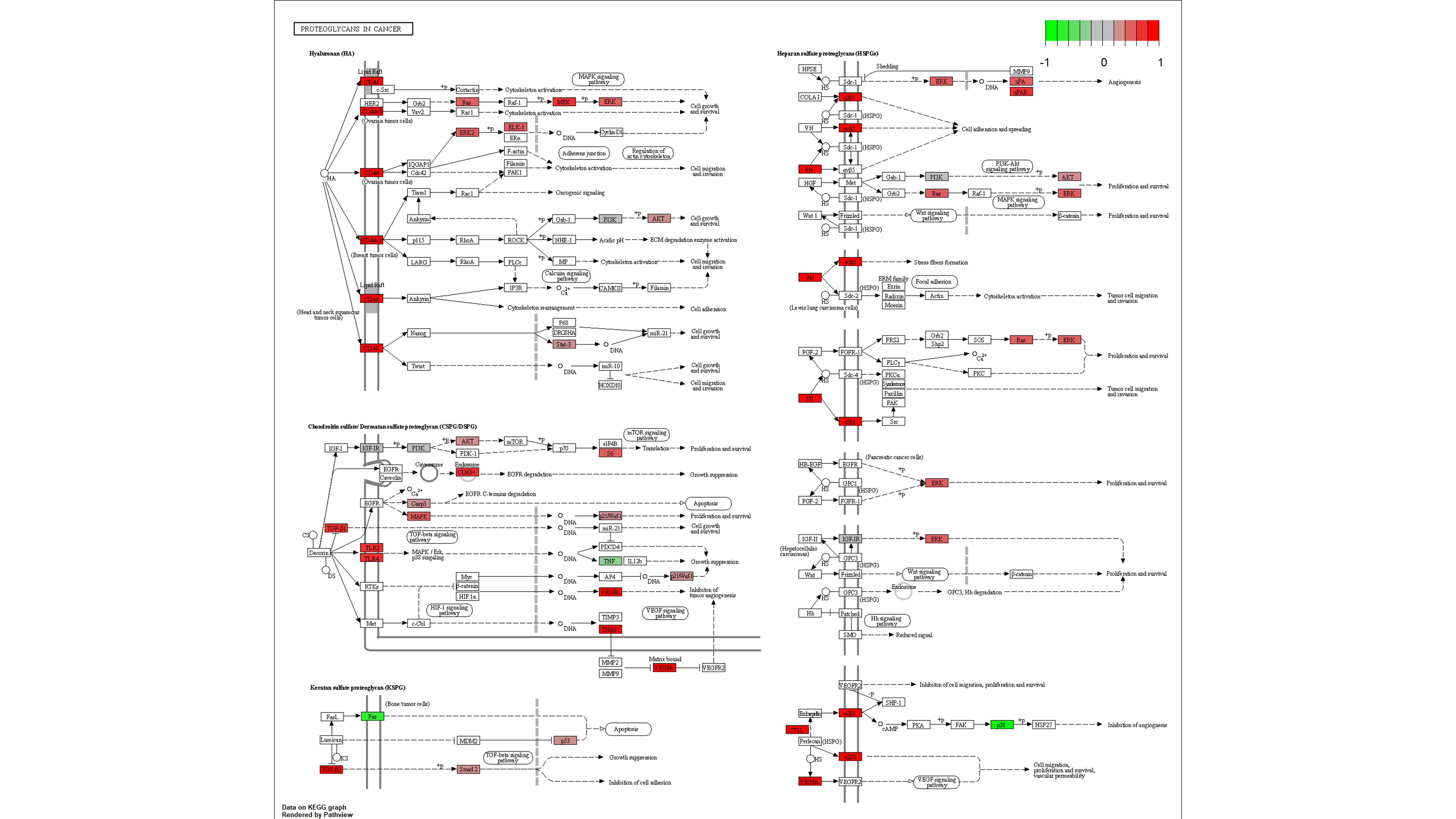


**Figure S4:** Gene set enrichment analysis of differential expressed genes for proteoglycans.


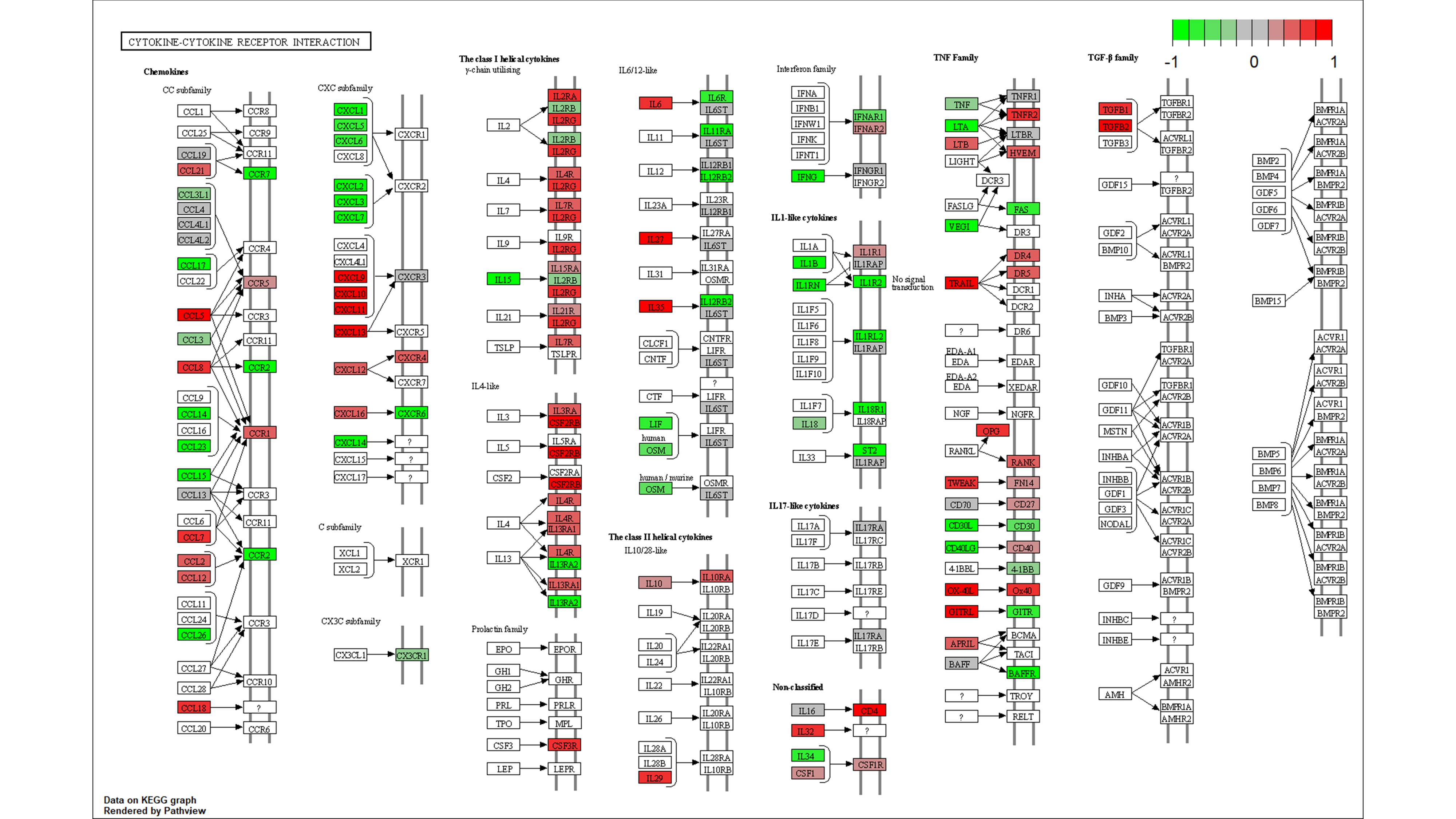


**Figure S5.** Gene set enrichment analysis of differential expressed genes between EMM and SMM involved in cytokine-cytokine receptor interaction.
